# Group I and group II metabotropic glutamate receptors are upregulated in the synapses of infant rats prenatally exposed to valproic acid

**DOI:** 10.1007/s00213-023-06457-w

**Published:** 2023-09-14

**Authors:** Simona D’Antoni, Sara Schiavi, Valeria Buzzelli, Samuele Giuffrida, Alessandro Feo, Fabrizio Ascone, Carla Letizia Busceti, Ferdinando Nicoletti, Viviana Trezza, Maria Vincenza Catania

**Affiliations:** 1 Institute for Biomedical Research and Innovation, National Research Council (IRIB-CNR), Catania, Italy; 2 Department of Science, Section of Biomedical Sciences and Technologies, University “Roma Tre”, Rome, Italy; 3 IRCCS Neuromed, Pozzilli, Italy; 4 Department of Physiology and Pharmacology, Sapienza University, Rome, Italy; 5 Neuroendocrinology, Metabolism and Neuropharmacology Unit, IRCCS Fondazione Santa Lucia, Rome, Italy

**Keywords:** mGlu1 receptors, mGlu5 receptors, mGlu2/3 receptors, Valproic acid, Ultrasonic vocalization, Autism spectrum disorder, LY341495, MTEP

## Abstract

**Rationale:**

Autism spectrum disorder (ASD) is a neurodevelopmental disorder characterized by impaired social interaction and restricted/stereotyped behavior. Prenatal exposure to valproic acid (VPA) is associated with an increased risk of developing ASD in humans and autistic-like behaviors in rodents. Increasing evidence indicates that dysfunctions of glutamate receptors at synapses are associated with ASD. In the VPA rat model, an involvement of glutamate receptors in autism-like phenotypes has been suggested; however, few studies were carried out on metabotropic glutamate (mGlu) receptors.

**Objectives:**

We examined the protein expression levels of group I (mGlu1 and mGlu5) and group II (mGlu2/3) mGlu receptors in rats prenatally exposed to VPA and evaluated the effect of mGlu receptor modulation on an early autism-like phenotype in these animals.

**Methods:**

We used western blotting analysis on synaptosomes obtained from forebrain of control and VPA rats at different ages (postnatal day P13, 35, 90) and carried out ultrasonic vocalization (USV) emission test in infant control and VPA rats.

**Results:**

The expression levels of all these receptors were significantly increased in infant VPA rats. No changes were detected in adolescent and adult rats. An acute treatment with the preferential mGlu2/3 antagonist, LY341495, attenuated the impairment in the USV emission in VPA rats. No effect was observed after a treatment with the mGlu5 selective antagonist, MTEP.

**Conclusions:**

Our findings demonstrate that the expression of group I and group II mGlu receptors is upregulated at synapses of infant VPA rats and suggest that mGlu2/3 receptor modulation may have a therapeutic potential in ASD.

**Supplementary Information:**

The online version contains supplementary material available at 10.1007/s00213-023-06457-w.

## Introduction

Autism spectrum disorder (ASD) includes a group of clinically and genetically heterogeneous debilitating neurodevelopmental disorders commonly characterized by impaired social interaction and stereotyped behaviors (Lord et al. 2020). Patients frequently exhibit intellectual disability, epilepsy, attention deficit hyperactivity disorder, and anxiety. The underlying cause is mostly unknown while about 20% of cases have a genetic cause, including chromosomal rearrangements, copy number variations, or point mutations (Abrahams and Geschwind 2008). Exposure to environmental factors during pregnancy may also play an important role in the etiology of autism and can increase the risk of developing autism (for a review, see Sealey et al. 2016). Thus, a combination of genetic, epigenetic, and environmental factors is believed to play a role in the etiology of autism. The neurophysiological bases of ASD are still unclear. There is currently no drug therapy that targets the pathophysiological mechanisms underlying the core symptoms of autism. The pharmacological approach is mainly symptomatic, aimed at favoring more adequate and socially acceptable behaviors, or aimed at limiting associated manifestations in comorbidities.

Clinical studies reported that maternal use of valproic acid (VPA), a common antiepileptic drug and mood stabilizer (Löscher 2002), can affect fetal brain development during pregnancy and induce several abnormalities in the exposed children (Christensen et al. 2013). Prenatal VPA exposure is considered an environmental risk factor involved in the pathogenesis of ASD in humans, and rats prenatally exposed to VPA represent a validated experimental model of autism (Nicolini and Fahnestock 2018; Tartaglione et al. 2019). Nevertheless, the mechanisms by which VPA exposure induces autistic-like phenotypes are still not clear.

Several studies examined the receptor expression profiles in different genetic and environmental animal models of autism. Alterations and imbalance of several neurochemical systems seem to be involved in ASD pathophysiology (Rubenstein and Merzenich 2003). Patients with ASD, as well as animal models of the disease, show altered expression of synaptic proteins (Zoghbi and Bear 2012) and functional defects in excitatory or inhibitory neurons (Gao and Penzes 2015; Howell and Smith 2019). Glutamate is the major excitatory neurotransmitter in the brain and is involved in a wide range of physiological functions of the central nervous system. Its action is mediated by ionotropic and metabotropic glutamate (mGlu) receptors (Reiner and Levitz 2018). Ionotropic glutamate receptors are multimeric ion channels that mediate fast synaptic transmission and include N-methyl-D-aspartate (NMDA) receptors, α-amino-3-hydroxy-5-methylisoxazole-4-propionic acid (AMPA) receptors, and kainate (KA) receptors (Traynelis et al. 2010). mGlu receptors are G protein-coupled receptors forming a family of eight subtypes (mGlu1 to mGlu8), of which mGlu1 and mGlu5 (group I) are coupled to Gq/11, and all other subtypes (mGlu2 and mGlu3, group II; mGlu4, mGlu6, mGlu7, mGlu8, group III) are coupled to G_i/o_ (Nicoletti et al. 2011). Growing evidence supports a role for mGlu receptor dysfunction in ASD (Nisar et al. 2022), and a disrupted mGlu receptor signaling, particularly mGlu5 receptor-mediated signaling, can be common to different forms of ASD (Bear et al. 2004; D’Antoni et al. 2014; Pignatelli et al. 2014; Petrelli and Bezzi 2018).

Changes in the expression of genes encoding proteins implicated in glutamatergic (excitatory) and GABAergic (inhibitory) neurotransmission and an excitatory/inhibitory imbalance were detected in VPA-exposed rats (Kim et al. 2014, 2016; Lenart et al. 2020). Alterations in synaptic transmission, connectivity, and synaptic plasticity have also been found in this animal model. VPA-exposed rats show an increase in glutamatergic activity (Tyzio et al. 2014) and in the long-term enhancement (LTP) of the thalamo-amygdala synapses (Lin et al. 2013), an altered expression of NMDA and AMPA ionotropic receptors (Rinaldi et al. 2007; Walcott et al. 2011; Martin and Manzoni 2014; Kim et al. 2016; Chau et al. 2017), and a dysregulation of the signal transduction pathways mediated by the activation of the ionotropic and metabotropic glutamate receptors (Kim et al. 2016, 2017). Compared to the numerous data on ionotropic glutamate receptors, few studies have been performed on mGlu receptors in this animal model (Chen et al. 2014; Peralta et al. 2016; Kim et al. 2016; Galineau et al. 2023).

In this work, we evaluated protein expression levels of group I and group II mGlu receptors in synaptosomes obtained from control and VPA-exposed rats at different ages. We observed an enhancement of both group I and group II mGlu receptor expressions in infant rats prenatally exposed to VPA and a correction of an early autism-like phenotype after systemic treatment with the preferential mGlu2/3 receptor antagonist LY341495.

## Methods

### Animals

We used Wistar rats (Charles River, Calco, Como). The female rats, arrived and housed in the animal facility, were mated with the males for a week. A vaginal smear was performed every day to check for sperm and evaluate the various phases of the estrous cycle. In the event of a positive outcome (presence of spermatozoa in the slide), the female rats were isolated from the male and placed inside new Makrolon cages (40 (length) × 26 (width) × 20 (height) cm), under controlled conditions (temperature 20–21 °C, 55–65% relative humidity, and 12/12 h light cycle with lights on at 07:00 h). Isolation day was considered gestational day 1 ( GD1). One day after birth (P1), the offspring were reduced to a number of 8 for each litter (2 females and 6 males). The animals were fed a standard diet, and food and water were made available ad libitum. Behavioral test was performed during the light phase of the cycle, between 10:00 and 15:00.

All procedures used comply with the guidelines dictated by the Italian Ministry of Health (Legislative Decree 26/2014) and by the European Community (Directive 2010/63/EU).

### Drug administration

Valproic acid (VPA) (Cayman Chemical) was dissolved in saline at concentration of 250 mg/ml. At GD 12.5, pregnant rats were treated intraperitoneally with 500 mg/kg VPA, a dose that induces autistic-like behavioral changes in the rat offspring (Servadio et al. 2016; Melancia et al. 2018). Control dams were treated with saline (SAL) solution. The offspring prenatally exposed to either VPA or SAL were treated with LY341495 (Tocris), MTEP (Tocris), or their vehicle (saline solution, VEH). Intraperitoneal injections of saline, LY341495 (1 mg/kg), or MTEP (0.3 and 1 mg/kg) were performed 1 h before the behavioral test.

### Synaptosome isolation

Frozen forebrains from control and VPA-exposed rats at postnatal day (P)13, P35, and P90 were allowed to thaw on ice, weighed, and homogenized in 10% (w/v) 0.32 M sucrose buffer containing ethylenediaminetetraacetic acid (EDTA 1 mM, Sigma), Tris–HCl (10 mM, pH 7.4, Sigma), phenylmethanesulfonyl fluoride (PMSF 0.5 mM, Sigma), and protease inhibitor cocktail (Roche). Nuclear fraction and cell debris were pelleted by centrifugation at 1000 × *g* for 10 min (4 °C). The supernatant was then centrifuged for 30 min at 10,000 × *g* (4 °C), and the resulting pellet was resuspended with the 0.32 M sucrose buffer, layered on top of discontinuous sucrose gradient (0.8, 1.0, and 1.2 M) and centrifuged for 2 h at 75,000 × *g* (4 °C). Synaptosomes were collected at the 1/1.2 M sucrose interface, diluted 1:2 in cold 0.32 M sucrose buffer, and pelleted (40,000 × *g*; 1 h; 4 °C). The pellets were resuspended in 40 mM Tris–HCl, pH 6.8, containing 0.5 mM PMSF and protease inhibitor cocktail. Protein concentration was calculated by BCA method (Pierce).

### Western blot analysis

Proteins were denatured in denaturating sample buffer (2X) at 37 °C for 5 min, resolved through 7.5% SDS–polyacrylamide gels (Bio-Rad), and then transferred onto nitrocellulose membranes (Hybond-C Extra, 0.45 μM, Amersham Biosciences) with a trans-blot semi-dry transfer cell (Bio-Rad). Filters were processed as indicated by the manufacturer of WesternBreeze Chemiluminescent Immunodetection System kit (Invitrogen). In brief, filters were blocked for 30 min with blocking solution and then incubated overnight at 4 °C with the following primary antibodies: anti-mGlu1α (mouse, 1:1000, G209-488 BD Pharmingen), anti-mGlu5 (rabbit, 1:2000, ab76316 Abcam), anti mGlu2/3 (rabbit, 1:1000, AB1553 Chemicon), anti-mGlu3 (rabbit, 1:500, AGC-012 Alomone Lab), anti-mGlu3 (rabbit, 1:1000, ab140741 Abcam), anti-mGlu3 (rabbit, 1:100, sc-271899 Santa Cruz), anti-mGlu2 (rabbit, 1:500, AGC-011 Alomone Lab), anti-mGlu2 (rabbit, 1:1000, ab150387 Abcam), anti-pan Homer (H-311) (rabbit, 1:1000, sc-15321 Santa Cruz), anti-Homer 1a (goat, 1:1000, sc-8922 Santa Cruz), anti-Norbin (mouse, 1:700, ab88877 Abcam), anti-β-actin (8H10D10) (mouse, 1:1000, #3700 Cell Signaling), and anti-GFAP (mouse, 1:100, MAB360 Millipore). After washing with TBST (Tris (100 mM, Sigma), sodium chloride (NaCl, 0.9%, Sigma), and Tween 20 (1%, Sigma)), filters were incubated with alkaline phosphatase-conjugated secondary anti-rabbit antibodies from Invitrogen kit at room temperature. Chemiluminescence was detected and quantified by computer-assisted densitometry, using the VersaDoc 4000 Imaging System (Bio-Rad). Values were normalized to those of actin. For each protein of interest, four to eleven samples from different animals were used. Data are expressed as percentage of controls.

The specificity of anti-mGlu receptor antibodies was tested by using brain tissue from mice lacking mGlu1, mGlu2, mGlu3, and both mGlu2 and mGlu3 receptors, available at IRCCS Neuromed (Pozzilli, Italy).

### Isolation‐induced ultrasonic vocalizations

On P9, the isolation‐induced ultrasonic vocalizations (USVs) emitted by each pup removed from the nest and placed into a Plexiglas arena were detected for 3 min by an ultrasound microphone (Avisoft Bioacoustics) sensitive to frequencies between 10 and 200 kHz. USVs were analyzed quantitatively using a Avisoft Recorder software (version 5.1).

### Statistical analysis

GraphPad Prism 9.5 software (GraphPad Software) was used for the statistical analyses. Statistical significance was determined using Student’s *t*-test (for western blot experiments), or two-way ANOVA followed by Newman–Keuls post hoc test (for USV analysis). Student’s *t*-test was used to compare the levels of proteins expression between the VPA-exposed rats and their respective controls at each age. Statistical tests and results of statistical analyses are specified in each figure legend. All data are presented as mean ± SEM.

## Results

### mGlu1 and mGlu5 receptors are upregulated in the synapses of infant VPA-exposed rats

ASD is considered a synaptic disorder (Ebrahimi-Fakhari and Sahin 2015). Thus, we performed the experiments on synaptosomes obtained from the forebrain of control and VPA-exposed rats. To examine whether age-specific changes in mGlu receptor expression occur in rats prenatally exposed to VPA, we performed our analysis at P13, P35, and P90, which are considered equivalent to human infancy, adolescence, and adulthood. We used only male offspring, because in the VPA model, the autistic-like phenotype is more evident in male offspring (Melancia et al. 2018).

**Fig. 1:**
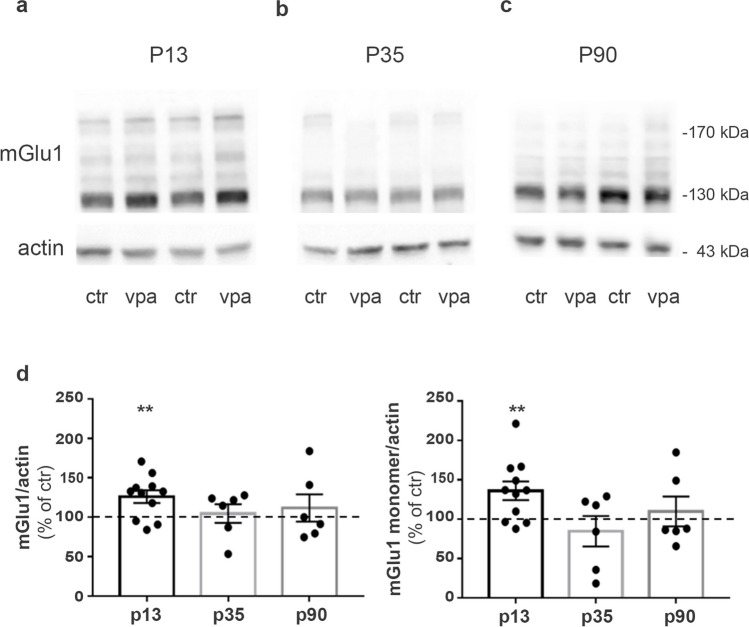
mGlu1 receptor expression is increased in rats prenatally exposed to VPA at postnatal day (P)13. **a–c** Western blotting of synaptosomes obtained from forebrains of control and VPA rats at P13 (**a**), P35 (**b**), and P90 (**b**). 30 μg of protein for lane was loaded. **d** Quantification of mGlu1 receptor expression levels normalized on actin (monomer and dimer on the left; monomer only on the right). Mean ± SEM. Data are expressed as percentage of respective controls. **p < 0.01 versus respective controls by unpaired *t*-test

**Fig. 2:**
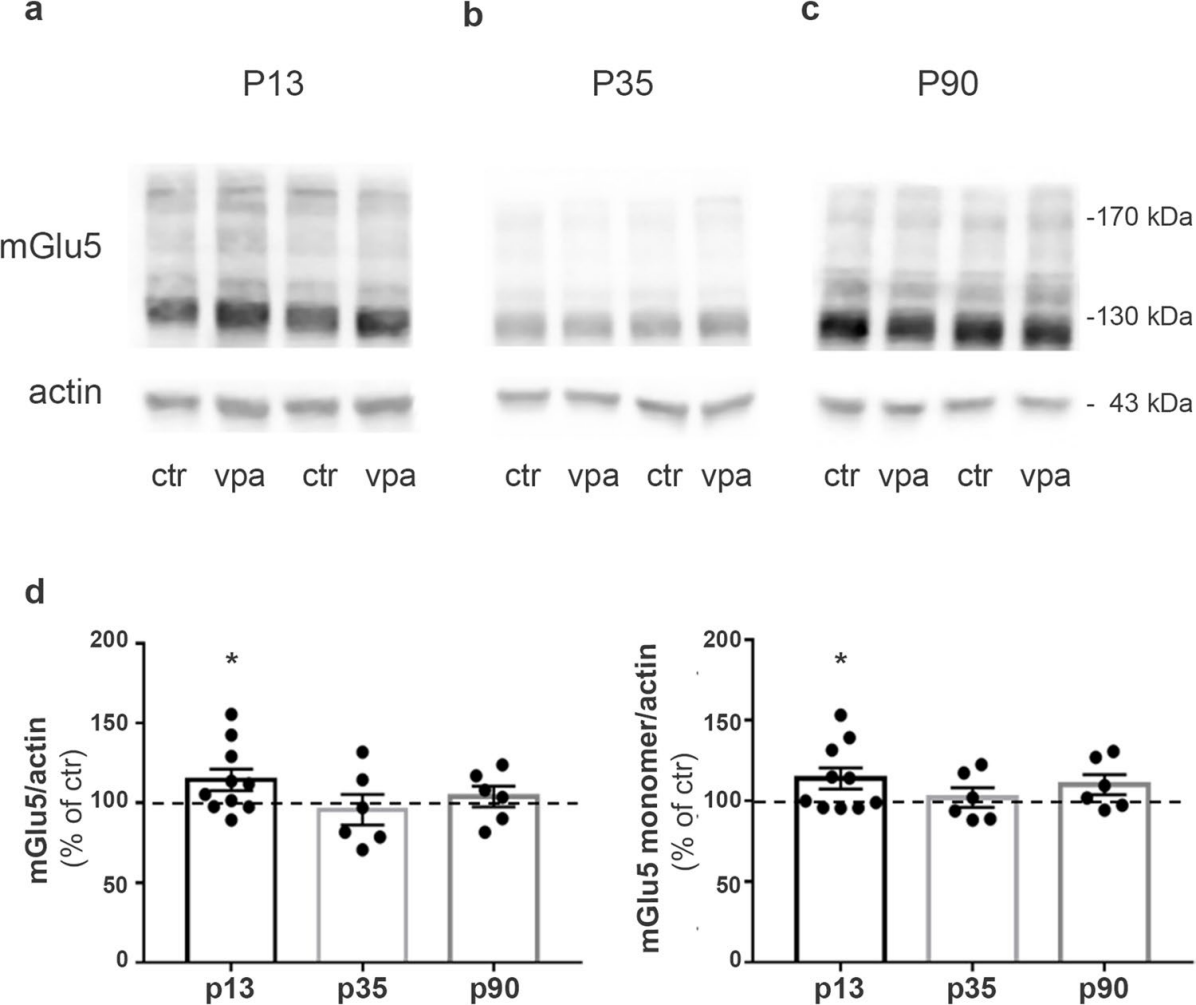
mGlu5 receptor expression is increased in rats prenatally exposed to VPA at P13. **a–c** Western blotting of synaptosomes obtained from forebrains of control and VPA rats at P13 (**a**), P35 (**b**), and P90 (**c**). 30 μg of protein for lane was loaded. **d** Quantification of mGlu5 receptor expression levels normalized on actin (monomer and dimer on the left; monomer only on the right). Mean ± SEM. Data are expressed as percentage of respective controls. *p < 0.05 versus respective controls by unpaired *t*-test

Western blot experiments showed an increase in mGlu1α receptor protein levels in the forebrain of VPA-exposed rats compared to their respective controls at P13 (Fig. 1a, d), whereas no changes between control and VPA-exposed rats were observed at P35 and P90 (Fig. 1b–d). Enhanced mGlu5 receptor protein levels could also be detected in VPA-exposed rats at P13, but not at later ages (Fig. 2) (see supplementary Fig. 1a for the specificity of anti-mGlu1α antibody). The specificity of anti mGlu5 antibodies has been previously tested using the cerebral cortex of mGlu5 knockout (KO) mice (Di Menna et al. 2023).

**Fig. 3:**
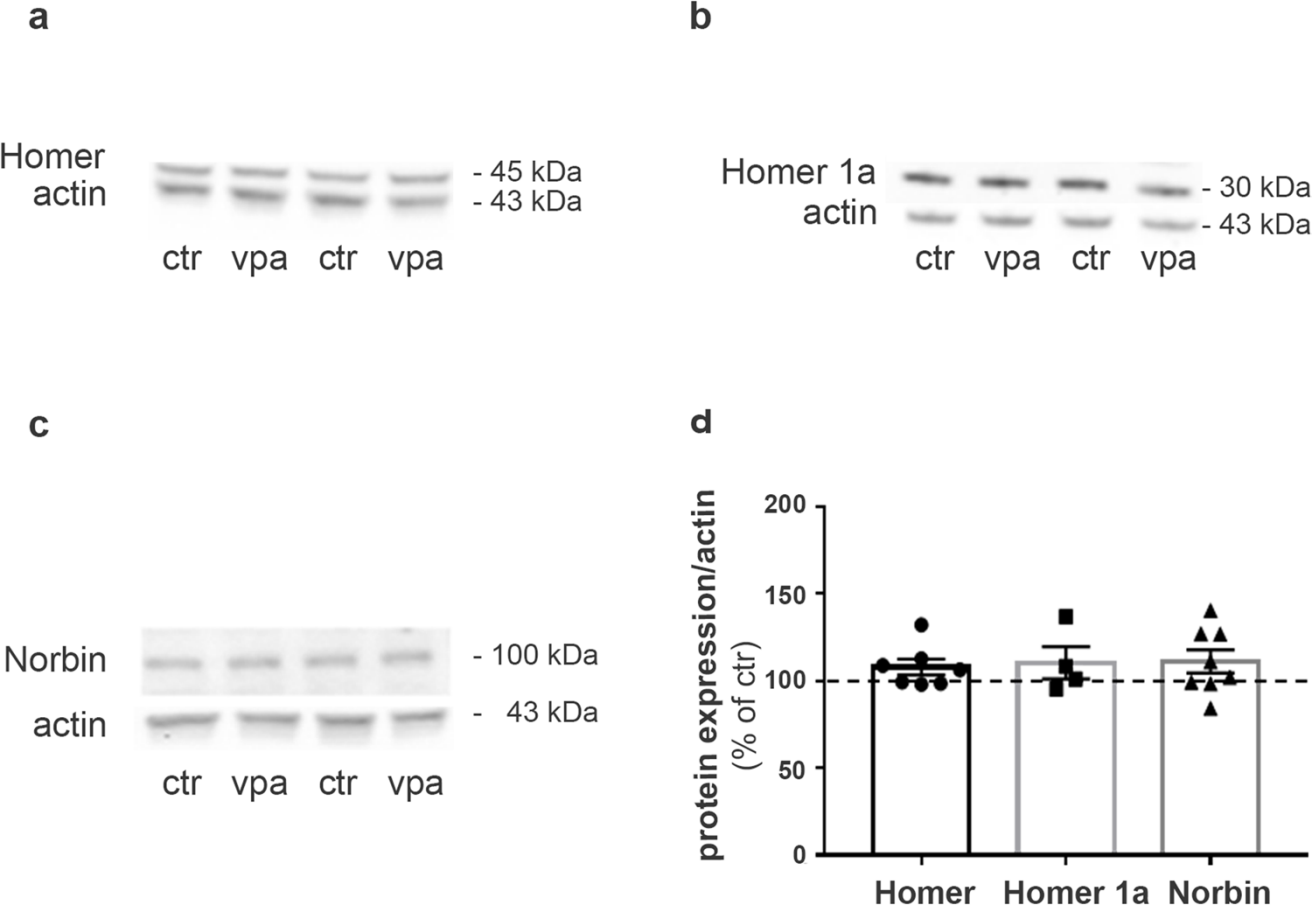
Homer, Homer 1a, and Norbin expression levels are similar in control and VPA-exposed rats at P13. **a–c** Western blotting showing expression levels of Homer (**a**), Homer 1a (**b**), and Norbin (**c**) in synaptosomes obtained from forebrains of control and VPA rats at P13. 30 μg of proteins for lane was loaded. **d** Quantification of Homer, Homer 1a, and Norbin expression levels normalized on actin. Mean ± SEM. Data are expressed as percentage of respective controls

Several properties of mGlu5 receptors depend on their physical interaction with scaffolding proteins, including Homer proteins (Kammermeier et al. 2000; Ango et al. 2001; Park et al. 2008) and Norbin (Wang et al. 2009). To study whether altered levels of mGlu5 were associated with changes in these proteins, we evaluated the expression of Homer, Homer 1a, and Norbin at P13 but observed no differences between control and VPA rats (Fig. 3).

### mGlu2/3 receptor expression is increased in synaptosomes of VPA-exposed rats at postnatal day 13

**Fig. 4:**
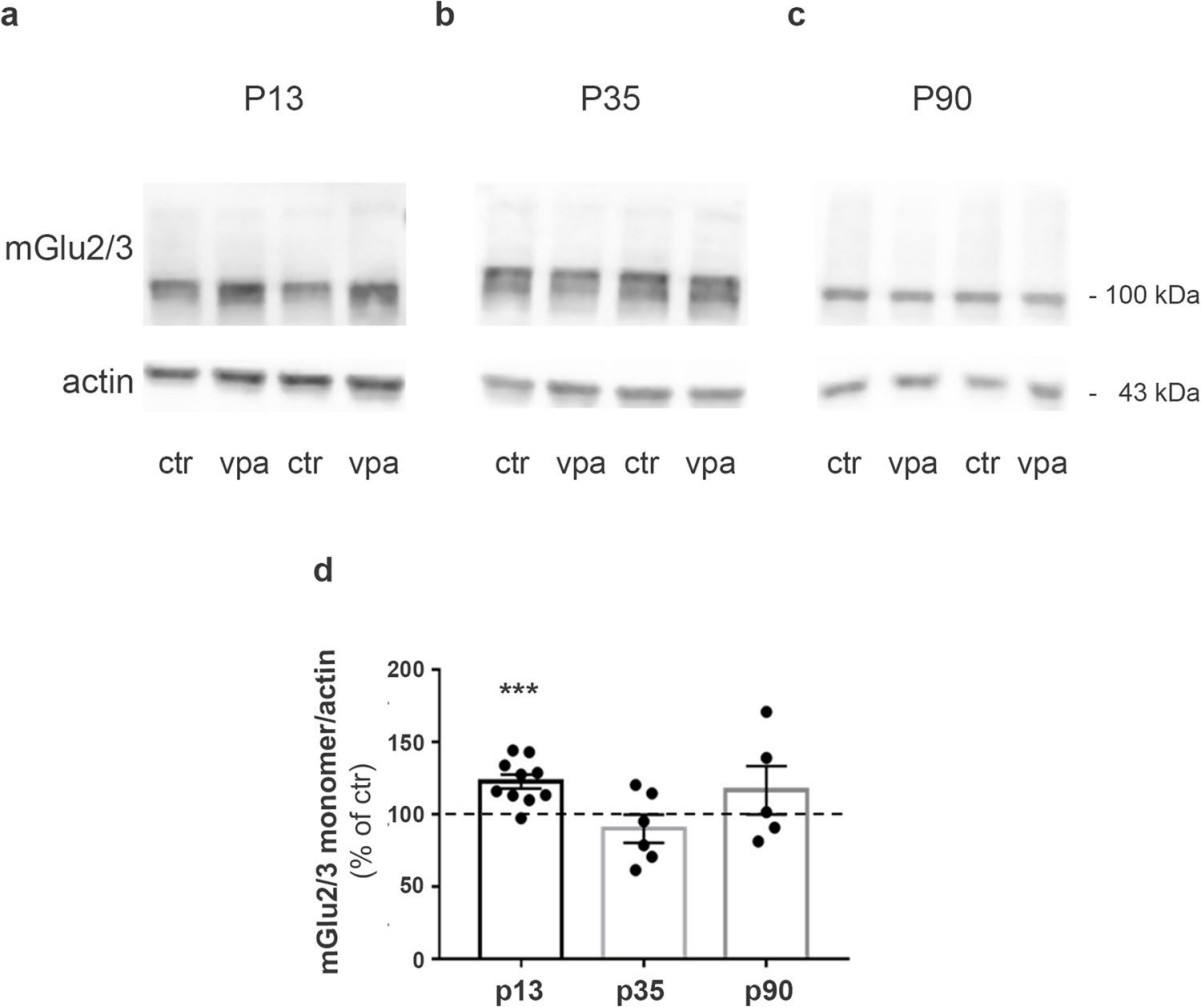
mGlu2/3 receptor expression is increased in infant rats prenatally exposed to VPA. **a–c** Western blotting of synaptosomes obtained from forebrains of control and VPA rats at P13 (**a**), P35 (**b**), and P90 (**c**). 30 μg of protein for lane was loaded. **d** Quantification of mGlu2/3 receptor expression levels normalized on actin. The level of dimers was too low to be accurately quantified. Mean ± SEM. Data are expressed as percentage of respective controls. ***p < 0.001 versus respective controls by unpaired *t*-test

For the study of group II mGlu receptors, we first used an antibody that cannot distinguish between mGlu2 and mGlu3 receptors. We observed an increase in mGlu2/3 receptor protein levels in synaptosomes isolated from the forebrain of P13 male rats prenatally exposed to VPA (Fig. 4a, d). No differences were found between control and VPA-exposed rats at P35 and P90 (Fig. 4b–d). The specificity of the antibody was confirmed by western blot experiment performed on lysates of prefrontal cortex obtained from mGlu2/3 receptor KO mice. As shown in the supplementary Fig. 1b, the anti‐mGlu2/3 antibody failed to recognize the bands corresponding to mGlu2/3 monomers and dimers in lysates from mGlu2/3 KO mice.

**Fig. 5:**
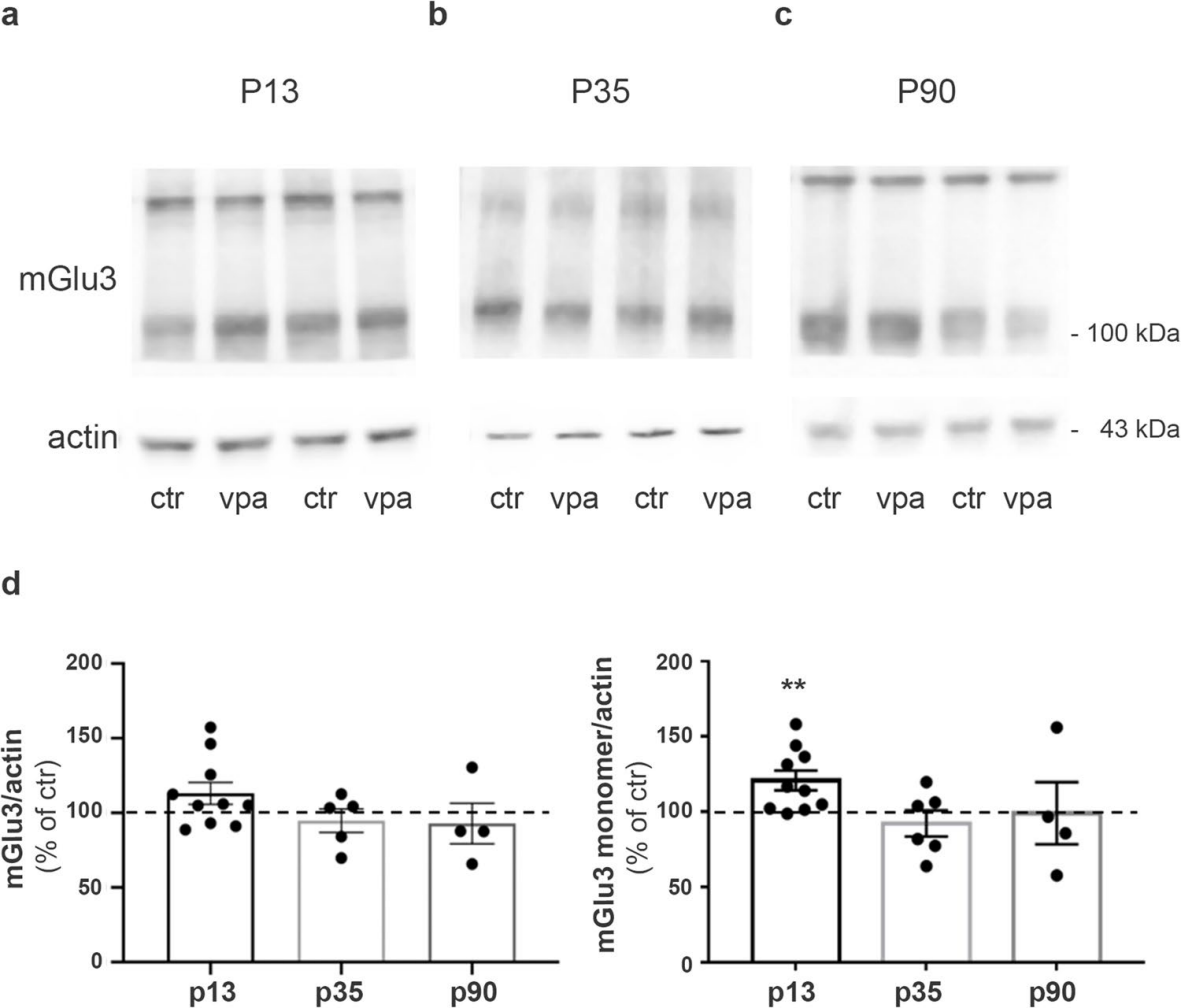
mGlu3 receptor expression is increased in rats prenatally exposed to VPA at P13. **a–c** Western blotting of synaptosomes obtained from forebrains of control and VPA rats at P13 (**a**), P35 (**b**), and P90 (**c**). 30 μg of protein for lane was loaded. **d** Quantification of mGlu3 receptor expression levels normalized on actin (monomer and dimer on the left; monomer only on the right). Mean ± SEM. Data are expressed as percentage of respective controls. **p < 0.01 versus respective controls by unpaired *t*-test

To establish whether the increase could be ascribed to either mGlu2 or mGlu3 receptors, we decided to examine expression levels of the two receptors separately. We could not study the expression of mGlu2 receptors because the commercially available antibodies that we have used (Abcam and Alomone Lab) labeled nonspecific bands that were still present in lysates from mGlu2 receptor KO mice (Supplementary Figure 1c). For mGlu3 receptors, we used three different antibodies (supplied by Abcam, Alomone Lab, and Santa Cruz), and we evaluated their specificity in western blot analysis on cortical homogenates from mGlu3 KO mice. We observed a specific signal only with the Alomone Lab antibody (Supplementary Figure 1d). Using this antibody, we found a significant increase in the expression levels of the monomeric form of mGlu3 receptors in VPA-exposed rats at P13 (Fig. 5a, d). No differences were detected between control and VPA-exposed rats at P35 and P90 (Fig. 5b–d).

mGlu3 and mGlu5 receptors are highly expressed in glial cells (D’Antoni et al. 2008; Spampinato et al. 2018), and an increased number of GFAP positive astrocytes have been detected in the rat VPA model (Deckmann et al. 2021). Since a small amount of the astrocytic processes associated with synapses is present in our synaptosomal preparation, we investigated whether the increased expression of mGlu2/3 receptors found in VPA-exposed rats at P13 was associated with changes in GFAP levels. At this age, GFAP levels did not differ between VPA-exposed and control rats (Supplementary Figure 2).

### Antagonism of mGlu2/3 receptors, but not mGlu5 receptors, corrects the reduced ultrasonic vocalization emission displayed by VPA-exposed infant rats

**Fig. 6:**
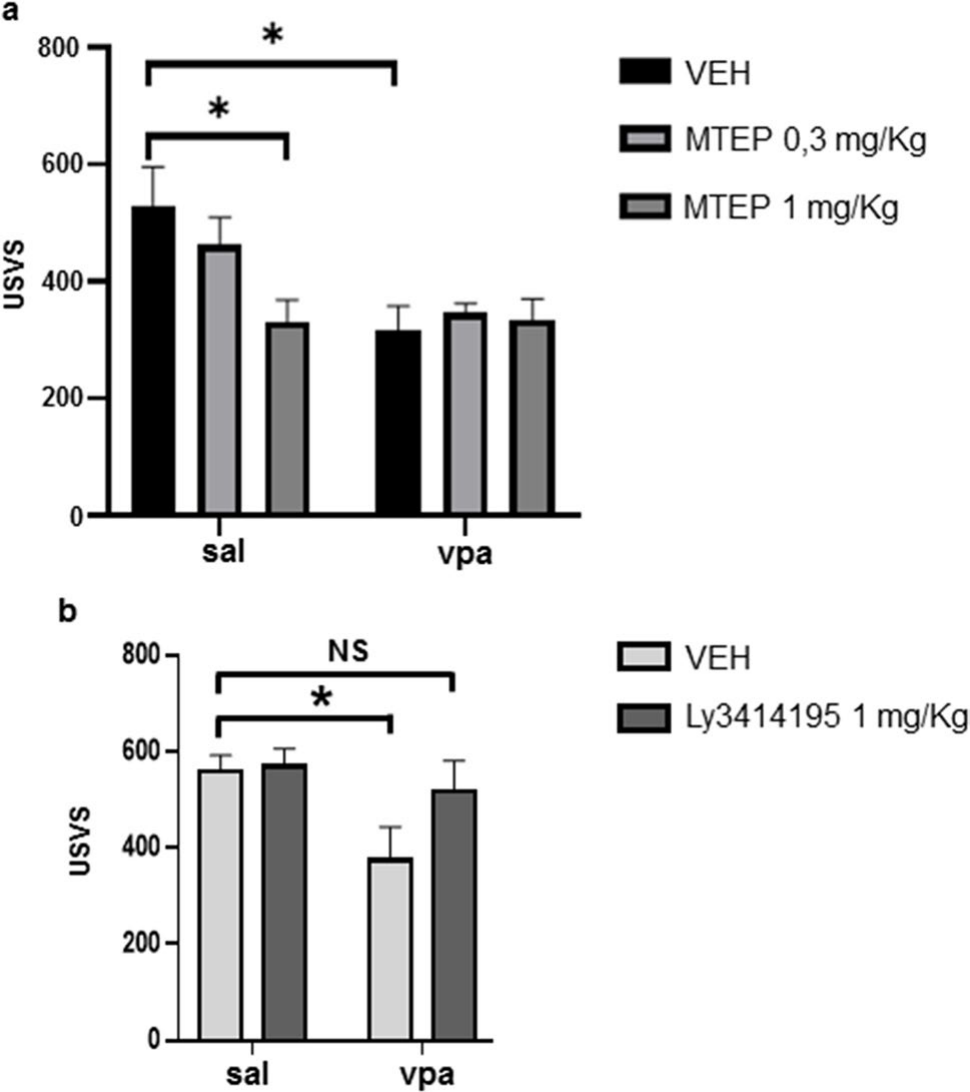
Acute treatment with Ly341495 mitigates the altered USV emission in VPA-exposed infant rats. No effects were detected after MTEP treatment. **a** Number of isolation-induced USVs in control and VPA-exposed-rats treated or not with MTEP. SAL VEH, *n* = 10; SAL MTEP 0.3 mg/kg, *n* = 10; SAL MTEP 1 mg/kg, *n* = 10; VPA VEH, *n* = 10; VPA MTEP 0.3 mg/kg, *n* = 10; VPA MTEP 1 mg/kg, *n* = 10. *p < 0.05 by 2-way ANOVA followed by Newman–Keuls post hoc test. **b** Number of USVs in control and VPA-exposed rats treated or not with Ly341495. SAL VEH, *n* = 12; SAL Ly341495, *n* = 10; VPA VEH, *n* = 11; VPA Ly341495, *n* = 8. *p < 0.05 by 2-way ANOVA followed by Newman–Keuls post hoc test

Evidence supports the use of mGlu2/3 and mGlu5 receptor modulators in the treatment of neurodevelopmental disorders (for a review, see Witkin et al. 2022). Thus, moving from the increased mGlu2/3 and mGlu5 protein levels found in VPA-exposed rats at P13, we decided to examine whether treatment with receptor antagonists could correct abnormal phenotypes of infant VPA-exposed rats. Socio-communicative symptoms were assessed through the ultrasonic vocalization (USV) test. In rodents, USVs play a fundamental role in mother–offspring interactions, as they induce maternal retrieval and elicit care giving behaviors in the dam (Servadio et al. 2015). We focused on mGlu5 and mGlu2/3 receptors because modulation of these receptors has been reported to correct repetitive behavior and social impairment in the VPA model (Mehta et al. 2011; Chen et al. 2014), while the effects of blocking the mGlu5 and mGlu2/3 receptors on USVs have not been examined. Control and VPA-exposed rats (P9) were treated 1 h before the test with MTEP (0.3 and 1 mg/kg), a selective negative allosteric modulator (NAM) of mGlu5 receptors. VPA-exposed male pups vocalized significantly less compared to the control group when isolated from the dam and siblings, as expected. A treatment with MTEP (1 mg/kg) caused a reduction of USVs in control rats (Fig. 6a), whereas it failed to affect USVs in VPA-exposed rats (Fig. 6a).

To block the activity of group II mGlu receptors, we used LY341495, a drug that preferentially antagonizes mGlu2 and mGlu3 receptors (reviewed by Schoepp et al. 1999). Systemic treatment with LY341495 (1 mg/kg, 1 h before the test) did not affect USVs in control rats and mitigated the reduced USV emission in VPA-exposed infant rats, although this effect was not statistically significant. Interestingly, after treatment with LY341495, there was no significant difference between control and VPA-treated rats on vocalization (Fig. 6b).

## Discussion

Here, we found an increase of mGlu1, mGlu5, and mGlu2/3 receptor expressions in the synapses of infant rats prenatally exposed to VPA. These differences disappeared in adolescent and adult rats, suggesting that the upregulation of these receptors occurring in the early life is corrected or compensated over the course of postnatal development. Alterations in neuronal excitability, connectivity, and synaptic plasticity were found in VPA-exposed rats during infancy (Rinaldi et al. 2007; 2008; Walcott et al. 2011). It will be interesting to examine the causal relationship between these changes and the upregulation of mGlu1, mGlu5, and mGlu2/3 receptors.

mGlu1 and mGlu5 show structural similarities but have a different brain distribution and exhibit different functional roles (Hermans and Challiss 2001). A limited number of studies have investigated a possible involvement of mGlu1 in ASD. An increased expression and activity of mGlu1 receptors have been found in the striatum of Shank2 KO mice (Modi et al. 2018), and a correction of repetitive behavior (reduced marble burying) has been reported in the fragile X syndrome mouse model after administration of the mGlu1 negative allosteric modulator, JNJ16259685 (Thomas et al. 2012). Inhibition of mGlu1, as well as mGlu5, also reversed deficits in social interaction and marble burying caused by deficiency of the translation repressor eIF4E binding protein 2 (Aguilar-Valles et al. 2015). Previous studies carried out in VPA-exposed rats showed an upregulation of mGlu1 receptor mRNA in different brain regions (Schiavi et al. 2022) and an increase in mGlu1 receptor protein levels in the hilus of the dentate gyrus and in the CA1 alveus region of the hippocampus at P30 (Peralta et al. 2016), but not in the somatosensory cortex at P14 (Rinaldi et al. 2007). We found an increase in mGlu1α receptor protein levels in forebrain synaptosomes at P13, but not at P35 and P90. Taken together, these findings suggest that prenatal exposure to VPA induces age- and region-dependent changes in the expression of mGlu1 receptors and lay the groundwork for future studies on mGlu1 receptors in models of ASD.

Unlike mGlu1 receptor, the involvement of mGlu5 receptor in ASD has been extensively studied, and changes in mGlu5 receptor expression have been reported in ASD patients (Boer et al. 2008; Fatemi et al. 2011, 2018; Lohith et al. 2013; Brašić et al. 2021; Mody et al. 2021; Carey et al. 2022; Galineau et al. 2023) and in different animal models of autism (Giuffrida et al. 2005, Verpelli et al. 2011, D’Antoni et al. 2014; Pignatelli et al. 2014, Gogliotti et al. 2016; Lee et al. 2019; Carey et al. 2022; Matrisciano et al. 2022; Di Menna et al. 2023). Nevertheless, the expression of mGlu5 receptors has not been systematically and longitudinally studied in VPA-exposed rats. No changes in mGlu5 receptor expression were detected in the somatosensory cortex and the prefrontal cortex of 2-week-old offspring of VPA-treated dams (Rinaldi et al. 2007; Kim et al. 2016) and in the hippocampus of VPA-exposed rats at P30 (Peralta et al. 2016). In contrast, an increase in mGlu5 receptor expression was found in the prefrontal cortex of 4-week-old VPA-exposed rats (Kim et al. 2016) and in the retina of VPA-exposed mice at P30 (Guimarães-Souza et al. 2019). A recent [^18^F] FPEB positron emission tomography imaging study revealed an increased mGlu5 receptor density in the cortex, hippocampus, amygdala, and striatum and a reduced mGlu5 receptor density in the thalamus, mesencephalon, and cerebellum in P35 VPA-exposed rats (Galineau et al. 2023). Thus, the absence of changes that we detected at P35 or P90 in synaptosomes from forebrain does not exclude regional modifications of synaptic expression of mGlu5 receptors at later ages. The increased mGlu5 receptor expression found in synaptosomes of VPA-exposed rats at P13 is in line with the reported increase in cortical long-term microcircuit plasticity (Silva et al. 2009), a form of plasticity that involves mGlu5 receptors (Le Be and Markram 2006), in VPA-exposed rats at P12–P15.

An excitatory/inhibitory imbalance is a characteristic of ASD (Gao and Penzes 2015; Howell and Smith 2019) and has also been reported in VPA-exposed rats (Kim et al. 2014, 2016; Lenart et al. 2020). Furthermore, GABAergic neurotransmission is altered in VPA-exposed rats (Yang et al. 2021). It will be interesting to examine whether changes in GABAergic transmission are driven by mGlu1 or mGlu5 receptors expressed by interneurons.

It is known that the modulation of mGlu5 receptors improves the pathological phenotypes in several mouse models of autism (reviewed in D’Antoni et al. 2014; Aguilar-Valles et al. 2015; Gogliotti et al. 2016; Vicidomini et al. 2017). Pharmacological inhibition (or genetic deletion) of mGlu5 receptors rescues cognitive and social deficits in models of fragile X syndrome (FXS), the leading monogenic cause of ASD (Michalon et al. 2012, 2014; Gantois et al. 2013), and is also effective in other models of autism (Silverman et al. 2010, 2012; Tian et al. 2015; Aguilar-Valles et al. 2015; Matrisciano et al. 2022). Despite initial positive results and extensive preclinical research, clinical trials have failed to confirm beneficial effects in FXS patients (Jacquemont et al. 2011; Berry-Kravis et al. 2016; reviewed by Scharf et al. 2015; Emmitte 2017; Witkin et al. 2022). Further studies are needed to fully understand the lack of translation from preclinical studies to human trials in FXS patients (Berry-Kravis et al. 2018), from which the future development of drugs for other neurodevelopmental disorders could benefit.

Systemic treatment with the mGlu5 NAM, MPEP, in mice was found to reduce repetitive self-grooming and marble burying behaviors in the offspring of VPA-treated dams with no effect on anxiety-like behavior (Mehta et al. 2011) or social behavior (Kim et al. 2014). Because mGlu5 receptor protein levels were increased at P13, we examined the effect of mGlu5 receptor blockade on autism-like phenotype in VPA-exposed infant rats. We used MTEP because it is more potent, selective, and orally bioavailable than MPEP (Cosford et al. 2003). We observed that MTEP (1 mg/kg) caused a significant reduction of USVs in control mice; this finding is in line with data obtained by others and can be related to the anxiolytic-like effects of mGlu5 antagonists (Iijima and Chaki 2005; Hodgson et al 2008). In contrast, systemic treatment with MTEP failed to induce changes in USVs in VPA-exposed rats, suggesting that the upregulation of mGlu5 receptors is not causally related to this specific manifestation of autism-like behavior.

mGlu2 and mGlu3 receptors are coupled to G_i/o_ proteins and are mainly expressed on pre-synaptic terminals, where they negatively regulate glutamate and GABA release (reviewed by Nicoletti et al. 2011). However, mGlu3 receptors are also present postsynaptically, where they functionally interact with mGlu5 receptors. This interaction is involved in mechanisms of activity-dependent synaptic plasticity in the prefrontal cortex and hippocampus (Di Menna et al. 2018; Joffe et al. 2021; Dogra et al. 2021).

Group II mGlu receptors play a role in depression (for a review, see Chaki 2017), cognition, and neuropsychiatric disorders, such as schizophrenia (for reviews, see Harrison et al. 2008; Maksymetz et al. 2017), but have been scantly studied in neurodevelopmental disorders. A decreased expression of mGlu2 and mGlu3 receptors has been observed in a mouse model and in patients with Rett syndrome, whereas an increased expression of both mGlu2 and mGlu3 receptors has been reported in a mouse model of MECP2 duplication syndrome (Vermudez et al. 2022). Reduced mGlu2 receptor protein and mRNA levels were detected in the hippocampus of young mice (P48) subjected to 4 weeks postweaning social isolation and in the BTBR mice, an idiopathic model of ASD (Caruso et al. 2022). Reductions in mGlu2/3 receptor protein levels and mGlu2 receptor mRNA levels were found in the amygdala of the offspring of VPA-treated dams at P21 (Chen et al. 2014), whereas no changes in mGlu2/3 receptor protein levels were detected in VPA-treated mice at P30 (Wang et al. 2021). These results, combined with our findings, suggest that changes in group II mGlu receptor expression induced by prenatal exposure to VPA are age-dependent and occur in the early postnatal life.

Systemic treatment with the preferential mGlu2/3 receptor antagonist, LY341495, had a beneficial effect on the pathological phenotype in a mouse model of the FXS (Choi et al. 2011) and in a rodent model of the MECP2 duplication syndrome (Vermudez et al. 2022). Our evidence that low doses of LY341495 (1 mg/kg, 1 h before the test) enhanced USVs in VPA-exposed rats, but not in control rats, suggests that pharmacological blockade of mGlu2/3 receptors may correct some early behavioral abnormalities associated with ASD. Chen et al. reported that a chronic treatment with N-acetylcysteine improved social and anxiety behavior in VPA-exposed rats, possibly acting through the activation of mGlu2 receptors in the amygdala (Chen et al. 2014). Altogether, these data corroborate the view that both activation and inhibition of group II mGlu receptors may have a therapeutic potential, with different effects depending on age. Interestingly, TP0473292 (TS-161), a novel safe, well-tolerated, and orally bioavailable in humans orthosteric mGlu2/3 receptor antagonist prodrug, having antidepressant-like effects in animal models, is now ready for use in clinical studies for treatment of depression and potentially for other neuropsychiatric disorders (Watanabe et al. 2022; Inatani et al. 2023).

It has been reported that mGlu2/3 receptor antagonists modulate the activity of dopaminergic and serotonergic systems (for a review, see Chaki 2019), which are altered in VPA-exposed rats (Nakasato et al. 2008; Schiavi et al. 2019; and reviewed by Kuo and Liu 2022). On the other hand, mGlu3 activation stimulates the production/release of brain-derived neurotrophic factor (BDNF) (Suzuki et al. 2017), which is abnormal in the VPA model of ASD (Almeida et al. 2014; Fuentealba et al. 2019). Whether changes in monoaminergic neurotransmission and/or production of BDNF contribute to the “therapeutic” effect of mGlu2/3 receptor blockade in VPA-exposed rats remains to be determined.

In summary, our findings provide additional evidence that changes in mGlu receptor subtypes are associated with ASD and suggest that, at least in the VPA model, mGlu2 and/or mGlu3 receptors might be targeted by therapeutic intervention particularly in the early phases of postnatal development. This attractive hypothesis should be validated using mGlu2 or mGlu3 selective NAMS, or extending the study to the VPA model in mice, which allows to establish whether genetic deletion of one or both receptors affects the development of the autism-like phenotype induced by prenatal exposure to VPA. Furthermore, we cannot exclude that the upregulation of mGlu receptors during early development could cause behavioral and cognitive deficits associated with autism later in life. Further research should be performed to understand whether an early overexpression of these receptors at synapses can affect neuronal connectivity and influence the trajectory of the disease.

### Supplementary Information

Below is the link to the electronic supplementary material.

Supplementary file1 (DOCX 964 KB)
