# Correction to: Group I and group II metabotropic glutamate receptors are upregulated in the synapses of infant rats prenatally exposed to valproic acid

**DOI:** 10.1007/s00213-023-06494-5

**Published:** 2023-11-09

**Authors:** Simona D’Antoni, Sara Schiavi, Valeria Buzzelli, Samuele Giuffrida, Alessandro Feo, Fabrizio Ascone, Carla Letizia Busceti, Ferdinando Nicoletti, Viviana Trezza, Maria Vincenza Catania

**Affiliations:** 1 Institute for Biomedical Research and Innovation, National Research Council (IRIB-CNR), Catania, Italy; 2 Department of Science, Section of Biomedical Sciences and Technologies, University “Roma Tre”, Rome, Italy; 3 IRCCS Neuromed, Pozzilli, Italy; 4 Department of Physiology and Pharmacology, Sapienza University, Rome, Italy; 5 Neuroendocrinology, Metabolism and Neuropharmacology Unit, IRCCS Fondazione Santa Lucia, Rome, Italy

**Correction to: Psychopharmacology**

https://doi.org/10.1007/s00213-023-06457-w

The authors regret having incorrectly reported the codes of three antibodies.

The reported errors are listed as follows:

1. The correct code of the Chemicon anti mGlu2/3 antibody is AB1553, not AB1506 as erroneously reported in Methods section paragraph western blot analysis and in the supplementary Fig. 1.
  The codes of the Alamone antibodies were inverted:
2. The correct code of the Alamone anti mGlu2 antibody is AGC-011, not AGC012, as erroneously reported in Methods section paragraph western blot analysis and supplementary Fig. 1.
3. The correct code of the Alamone anti mGlu3 antibody is AGC-012, not AGC011, as erroneously reported in Methods section paragraph western blot analysis and supplementary Fig. 1.

The corrected supplementary figure is also reflected in the original article.

The authors state that these unintentional errors do not change the scientific conclusions of the article in any way and wish to apologize for any inconvenience caused.

The original article has been corrected.

