# Supplementary material for: Group I and group II metabotropic glutamate receptors are upregulated in the synapses of infant rats prenatally exposed to valproic acid
Source: Psychopharmacology (Berl). 2023 Sep 14;240(12):2617–29. doi: 10.1007/s00213-023-06457-w (PMC10640443; doi:10.1007/s00213-023-06457-w)

**Supplementary material**

**Manuscript title:** “Group I and group II metabotropic glutamate receptors are upregulated in the synapses of infant rats prenatally exposed to valproic acid”.

**Authors:** Simona D’Antoni, Sara Schiavi, Valeria Buzzelli, Samuele Giuffrida, Alessandro Feo, Fabrizio Ascone, Carla Letizia Busceti, Ferdinando Nicoletti, Viviana Trezza, Maria Vincenza Catania.

**Journal:** Psychopharmacology.

**Affiliation Corresponding Author:** Institute for Biomedical Research and Innovation, National Research Council (IRIB-CNR), Catania, Italy.

**Corresponding author email:** mariavincenza.catania@cnr.it.

**Supplementary Figure 1**: Specificity of anti-mGlu receptor antibodies used for Western blotting experiments. **a**: Western blot showing the absence of signal in the cortex from mGlu1 KO mice. 50μg of proteins from cerebellar synaptosomes of wild type (WT) and *Fmr1* knockout (KO) mice and from cortex of mGluR1 KO mice was loaded. **b**: The image shows the absence of mGlu2/3 receptors in lysates obtained from mGlu2/3 KO mice. 20μg of proteins was loaded. Proteins were obtained from VPA rats (synaptosomal preparation), mGlu2 receptors KO mice, mGlu2/3 receptors KO mice and mGlu3 receptors KO mice. **c**: Images showing the presence of unspecific signals with antibodies anti-mGlu2 receptor supplied by Alamone Lab and Abcam. 20μg of proteins were loaded. Proteins was obtained from VPA rats (synaptosomal preparation), mGlu2 receptors KO mice, mGlu2/3 receptors KO mice and mGlu3 receptors KO mice. **d**: The anti‐mGlu3 antibody produced by Alamone Lab did not recognize the proteins corresponding to mGlu3 monomers and dimers in lysates from mGlu2/3 and mGlu3 KO mice. The antibodies supplied by Abcam and Santa Cruz show unspecific signals. Western blot experiments performed in synaptosomes of VPA exposed rat and in lysates obtained from mGlu3 receptor KO mice; mGlu2 receptor KO mice; and mGlu2/3 receptors KO mice. 20μg of proteins was loaded.


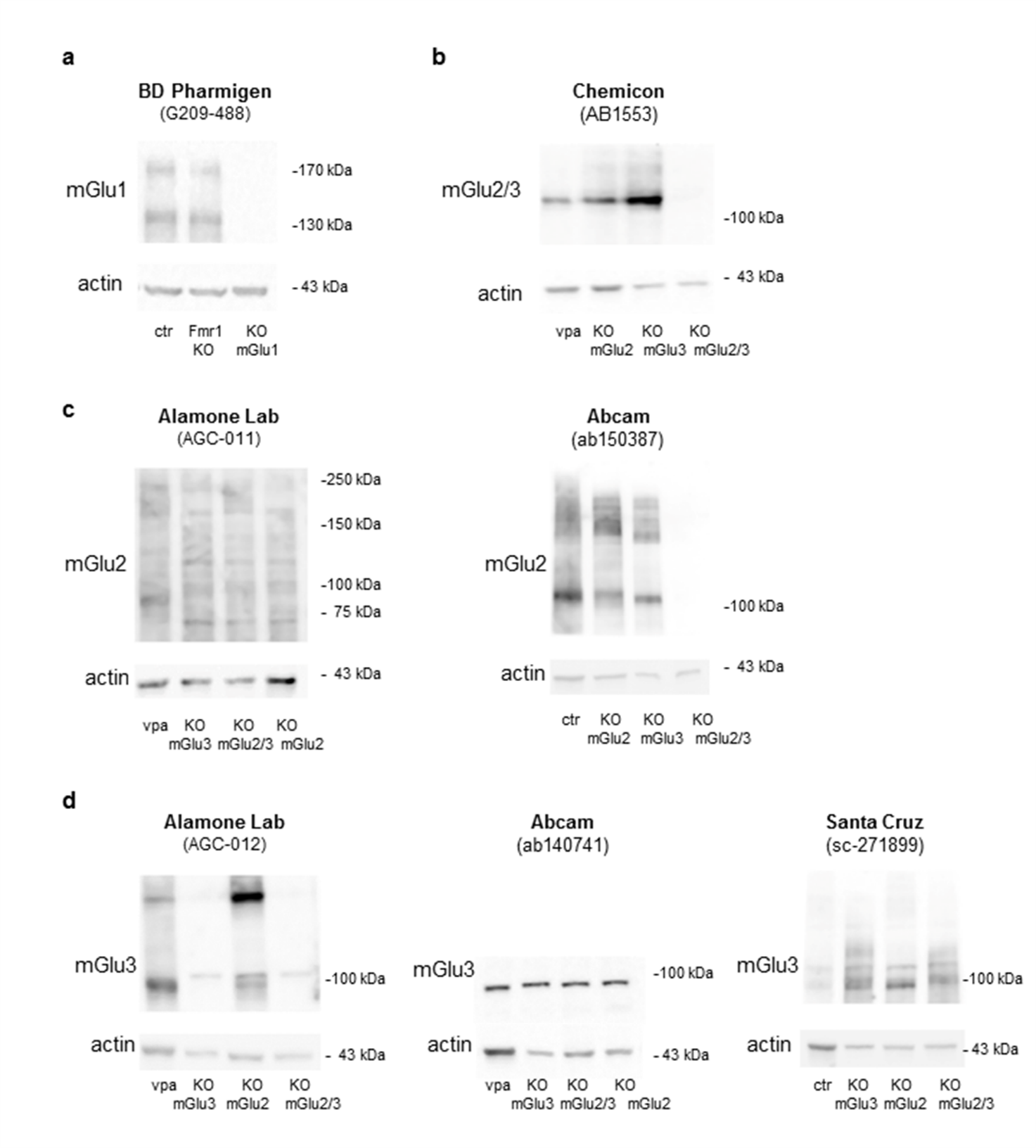


**Supplementary Figure 2: GFAP expression levels are similar in synaptosomes from control and VPA exposed rats at P13. a:** Western blotting of synaptosomes obtained from forebrains of control and VPA rats at P13. 30μg of proteins for lane was loaded. **b**: Quantification of GFAP expression levels normalized on actin. Mean + SEM. Data are expressed as percentage of controls.


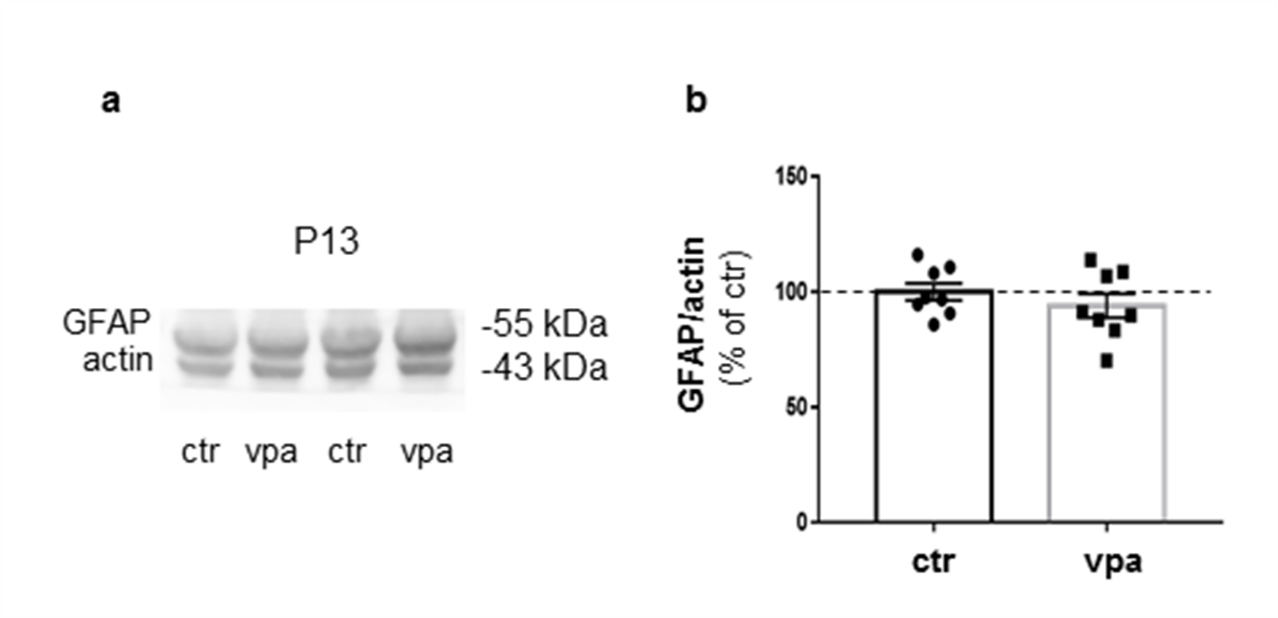

Supplement: Supplementary file 1 — Supplementary file1 (DOCX 964 KB) [file 213_2023_6457_MOESM1_ESM.docx]
